# Selections from the Foreign Periodicals

**Published:** 1847-01-01

**Authors:** 


### Periscope;

OR,

### CIRCUMSPECTIVE REVIEW.

### Selections from the Foreign Periodicals.

### On the Retrospective Semeiological Characteristics presented by the Nails. By J. Beau, M.D.

It is familiarly known that, after certain diseases, as severe fevers, the *nails* are sometimes cast as well as the hair. They are reproduced when the unguinal matrix resumes its secretory functions, and, after a certain time, the new nails have acquired the same form and consistence as the old ones. But less severe diseases, which do not lead to the actual shedding of the nail, may yet interfere with the secretory function sufficiently as to leave marks of their existence by certain traces on the nails. These are found in the form of furrows or depressions placed transversely on the back of the nail. They vary from the slightest depression to one which nearly occupies the whole substance of the nail. They are found larger upon the thumb than upon the finger nails, and sometimes it is upon these alone that their traces are to be discovered. They are of varying length and breadth. They will be found at different points of the nail according to the space of time which has elapsed since the occurrence of the malady. To ascertain this with exactitude, the rate of growth of the nail must be observed, which may be done by imprinting upon it an indelible mark, and observing how much this advances towards the free edge of the nail in a given time. Experiments of this kind shew that the nails of all the fingers increase a millimetre ($\frac{2}{5}$ of a line) per week; while the nails of the toes only grow thus much in four weeks. From this we may see that the thumb-nail, which, in an adult male is about 20 millimetres long, inclusive of the portion concealed in the matrix, requires 20 weeks, or 5 months, to perform its entire evolution, while that of the great toe, which averages about 24 millimetres, requires 96 weeks, or two years, for the same purpose. This law of increase is the same in health and disease, and growth differs only in the latter in the less abundance of the materials which are deposited. The furrows may be solitary, or, if there have been other illnesses, two or three are found upon the same nail, separated by more or less considerable spaces. As *typhoid fever* is a disease which may lead to a complete shedding of the nails, so also it is the chief of those in which these furrows are found. To this we may add the various pyrexiæ and phlegmasiæ; and all affections in which the reparative and assimilatory powers are suspended or notably diminished—especially when in such febrile affections are present. They are observed, also, after the operation of various moral causes which have markedly influenced the digestive functions. The few days abstinence after parturition frequently suffice to leave traces on the nails. Of course the grooves are deep only in proportion to the gravity of the affection, and unless a slight attack appear very suddenly, the edges of the depression will be scarcely perceptible.

As we have already noticed, it is upon the thumb and great toe nails we must search for these furrows; as, when existing, they are always found on these, and often upon them alone. To estimate the time which has elapsed *since the occurrence of the disease* giving rise to the furrow, we must count as many weeks as there are millimetres between the furrow and the posterior margin of the nail, bearing in mind that this is hidden to the extent of 3 millimetres by the epidermic fold which bounds the nail behind. These furrows of the thumb-nail however, can never furnish the indication of a disease having existed for a longer period than 5 months prior to the examination, but those of the great toe will furnish such as far back as two years—each millimetre there representing a month —its posterior border being, however, hidden by the epidermis to the extent of 5 millimetres. The *breadth* of the furrow indicates the *duration* of the disease, a millimetre expressing a week or a month, according as the thumb or great toe-nail is examined. If the disease has continued for less than a fortnight, and has only been slight, it leaves scarcely any traces on the toe-nail. Even the mode of the *invasion* and *termination* of the disease may be established ; for, if we find the edges of the furrow of the thumb-nail are sharp and decided, we know it has appeared and terminated suddenly, while, when this is not the case, the transition from health to disease has been gradual—always remembering that the anterior edge of the furrow is that which indicates the commencement of the disease, and the posterior one its termination.

It is not pretended that every acute disease must infallibly furnish these furrows. Exceptions do occur, but in spite of such, we believe these researches are sufficiently interesting to call for publication. In verifying them, practitioners will often observe with what extraordinary precision they can recognise the existence and circumstances of a past disease, to the utter astonishment of the patient. The study is therefore useful in a semeiological point of view, as it may also be in those cases of legal medicine in which it is the interest of an accused person to deny the former existence of disease. *Reil,* in his *Memorabilium Clinicorum,* notices the occasional formation of a white semicircular line after severe fever.—*Archives Generales,* T. xi, pp. 447-458.

---

### Blisters in Confluent Small-pox.

M. Piorry has for some time past derived great assistance from the use of blisters as a means of preventing the scarring of the face by the cicatrices of confluent small-pox. The pus, retained so long in contact with the tissues, and altered in character through the agency of the air which passes through the pustules by endosmosis, operates extensive local destruction, and proves very injurious to the system when re-absorbed. Various practitioners have proposed measures for obviating this inconvenience, as by cauterization of each pustule (impossible in the confluent disease), the opening them by scissors, needles, &c. Experience, however, shews that over such means the blister has the advantage of—1, opening at one time the whole of the pustules over which it is applied : 2, evacuating their entire contents, and preventing the consequences of the sojourn or resorption of pus : 3, counteracting the attendant erysipelas,by diminishing the swelling ; and 4, causing the scabs to fall much sooner from the face than from other parts of the body. It has an advantage over mercurial plaisters in not risking the excitement of salivation, the extent of evil which results from its use being a slight ischuria. The various plaisters applied as abortives in this disease have too been reproached with exerting a repellant action, and directing the morbid action upon the brain and its membranes. A blister, on the contrary, rather acts as a derivative—*Gazette des Hôpitaux,* No. 101.

[Our neighbours have shewn a laudable anxiety for the discovery of the means for the prevention of the hideous marks of the small-pox, once so common, now so rare. The very rarity of the occurrence has, however, led too little attention to be paid amongst ourselves for its prevention. We think M. Piorry's practice well worth a trial.—*Rev.*]

---

### Harden on Isopathia.

Dr. Harden of Liberty County, Georgia, has resumed (in the July Number of the *American Journal of Medical Sciences*) his observations upon *Isopathia* (that is upon diseases of identical nature, although presenting different external appearances), which we gave an account of in a former number (July 1845). He prefaces them with some strictures upon the existing systems of *Nosology*, as being too exclusive. The *etiological method* of Boerhaave and Hoffman is only applicable to such diseases whose causes are known—while identical diseases may be brought on by very different general causes acting upon a system with a certain predisposition which is not understood. So, in regard to the *symptomatological system* of Cullen, Sauvages, &c. many diseases distinct in their nature, when seated in the same organ, will manifest symptoms so identical as to mislead the wisest. "The same objection applies with still greater force to the *functional* or *physiological* plan of Dr. Good," and the *tissual, organic,* or *topographic* system of Bichat, more or less embraced by Broussais, Bouillaud, &c. The localization of all essential disease, in referring it entirely to an altered vital action, without regard to the mechanical or chemical actions of the system is necessarily defective. "As the same morbid action may attack different tissues or parts of the body, so it is equally true that different morbid actions may attack the same tissue or part of the system; and, although pathological formations or types are slightly different in various parts of the body, as is the case with animals or plants in different climates or soils, yet the species is invariable and always to be recognized."

*Pathological Anatomy* originating with Bonetus, Valsalva and Morgagni, and advanced by modern labours, such as those of Laennec, Cruveilhier, Hope, Carswell, &c., has better claims than the preceding systems, but still is defective. "*Pathological anatomy* is not *pathology,* the lesions *left after death are not disease,* but simply the effects of the morbid action which had been going on in the system during life, and should be considered as nothing more than a kind of special symptomatology." The revival of the *Humoral Pathology* under the auspices of Andral, Liebig, Dumas, Prout, Müller, &c. gives rise to the hope that disease will ere long be brought within the range of physical laws, and be treated upon physiological principles.

"The question now comes up, by what method are we to distinguish and classify diseases? Our answer emphatically is,—by a rightful combination and use of all the methods that have been passed in review, so far as they may be applicable to the case before us; by an investigation of the *causes,* so far as they may be known to us, whether predisposing or exciting; by a careful observation and comparison of *symptoms,* as they present themselves in connection with their *seats* or *organs,* whose functions may be disturbed during the progress of the disease; by a critical inspection of the *lesions* which may be presented after death; by an examination of the state of the *blood* or *other fluids;* and, lastly, by all those collateral aids which may be afforded by the *history* of the disease, and other circumstances alluded to in my former paper."

In the paper referred to Dr. Harden had, with the object of tracing the isopathic connection of various diseases, divided these into certain generic types; viz. Febrile, Inflammatory, Purulent, Tuberculous or Strumous, Scorbutic or Hæmorrhagic, Exanthematous, Hydropic, and Gouty or Podagric Types. In the present paper, of these, the Inflammatory and Purulent Types are treated of at considerable extent, with great lucidity and comprehensive power. The limited space we have at command quite prevents our doing justice to so elaborate a communication; and we prefer, therefore, deferring any notice of it until another opportunity, or until the completion and re-publication of the series of papers. We feel obliged by Dr. Harden's frequent and complimentary allusion to the *Medico-Chirurgical Review.* No exertions on our part will be wanting to furnish him and our other readers with an accurate and complete view of the progress of medical science.

---

### M. Malgaigne on Simple Inflammation or Pseudo-Strangulation of Hernias.

According to Boyer, strangulation consists in a more or less complete interception of the course of the fæcal matters, an interception, preventing the reduction of the hernia, and giving rise to the gravest consequences. This definition is imperfect, inasmuch as it excludes the strangulation of omental hernias. The same surgeon also states that a variety of strangulation consists "in the choking up (*engouement*) of the displaced intestine by fæcal matters accumulating in it; so that in old hernias collections of excrements are almost always found." M. Malgaigne's observations are quite opposed to these views, and he has cited, in his memoir upon the subject, the cases of a great number of persons having hernias, in whom the action of the bowels continued perfectly regular, and whose hernias percussion proved to be empty of fæcal matter. So also, in a great number of *post-mortems,* he never met with such accumulation, although the strangulated portion of the intestine was frequently of considerable length. The opinion is, he observes, manifestly erroneous, for such choking up must result from the accumulation of indurated excrements, while the immense majority of hernias, large and small, is constituted by the displacement of the small intestines, in which, as every one knows, excrements, and especially indurated ones, are not found. The indurated condition of some irreducible hernias has given rise to the error.

In the cases in which the large intestine is implicated in a hernia, such accumulation would seem natural enough, and yet M. M. sought, during two years, among all the published cases, as well as in many of those occurring in his own private or hospital practice, with the resnlt of discovering only one example of this. Rejecting, then, this doctrine as ill-founded and dangerous, M. M. states that, in the question of strangulation three occurrences should be borne in mind. 1. There may be simple strangulation, which is very rare, occurring without inflammation, and inducing gangrene in some hours. 2. Simple inflammation, which is very common, and almost always confined to the serous membrane of the hernia. 3. Inflammation of the mass of the contents of the hernia of the omentum, adipose tissue, and all the tunics of the intestine. This scarcely ever occurs but as a consequence of the former conditions, whether from the effect of the strangulation itself when this is not sufficiently severe to induce immediate gangrene, or from the injudicious employment of the taxis in instances of simple herniary peritonitis. It is the *herniary peritonitis* which constitutes what M. M. terms *pseudo-strangulation.*

Herniary Peritonitis may give rise to either the *adhesive* or the *suppurative* form of inflammation. The former is frequently slight and transient, revealing itself by more or less severe colics, which are relieved by rest, cataplasms, and hot drinks. It especially affects those in whom the hernia is badly supported, in damp changeable weather, and after excess at table or drinking, although this last circumstance is not so important as some think, inasmuch as subjects having the large hernias frequently commit every kind of debauchery with impunity.

The partaking of cabbages or beans often gives rise to an accumulation of gas and the production of great suffering in the subjects of hernia. Among the most common symptoms are dull colicky pains, meteorism, and a physical or mental uneasiness, especially after any but the most moderate meals. For a long period M. Malgaigne attributed this *malaise* to mere change in the position of the viscera, but he now considers it a symptom of peritonitis. In some cases the local and general uneasiness is very slight, but a difficulty is found in the reduction of the tumor. If we are called at the commencement of the case, we must at once reduce the hernia; but if we see it later, we must first relieve the irritation in order to facilitate the taxis, which however must not be too long delayed. In a yet farther advanced stage, there are pains in the abdomen and the tumor, constipation, and vomiting. In his memoir, M. Malgaigne has cited several facts, in which intense pains and a seeming irreducibility have yielded to a long-continued pressure. "The slightness and fugacity of the herniary colics, which I consider as the first stage of a peritonitis, have been objected to me," M. Malgaigne observes, "but it is to be noted that many pleural adhesions are met with in persons who have never suffered other than slight stitches in the side, and adhesions of the omentum to the hernial sac are observed in those who have never suffered any durable uneasiness in the hernia."

The following are the practical conclusions which M. Malgaigne draws from his experience. 1. In all old intestinal hernias, which have never been supported by a bandage, or for which the bandage has been long abandoned, there is no true strangulation—the ring or rings being much larger than is required for the pedicle of the hernia. This is the result of all the examinations I have made, whether upon the living or the dead; for I have never yet met with an exception. 2. In pure epiploceles, whatever may be their size, it is generally an adhesive or suppurative peritonitis which exists; and, although the reality of a strangulation in such cases cannot be absolutely denied, its demonstration remains to be made. I cannot avoid, however, remarking upon the inconsistency of those surgeons who, operating to remove a pretended strangulation of the omentum, and finding it changed in texture, apply a ligature to it, thereby submitting it to a strangulation ten times more severe than that for which the operation was conducted. 3. Consequently, in these two specified cases, an operation is always irrational, and should in future be abandoned by surgeons.

"If we adopt these conclusions, which then are the indications to be fulfilled? First of all it is essential to ascertain if the hernia could in part or entirely be returned before the accident, if the patient could return it himself, and retain it without uneasiness in the abdomen. For in this latter case we must refrain from the taxis, as we risk by succeeding in its use the production of an unexpected death. When the circumstances are favorable, and we are called in good time, before the skin of the scrotum participates in the inflammation, the taxis is the first of all means. If it does not succeed at first we may cover the tumor with a cataplasm, place the patient in a bath, give a tobacco injection, and then recur to it. This procedure is founded upon the fact that, frequently the inflammation appears to result from a simple displacement of the viscera, when their reduction suffices to dissipate it. But I cannot too strongly recommend the most gentle manners of proceeding, and that the surgeon should always bear in mind that he has to do, not with choked up organs, but with inflamed tissues; and, if he finds resistance, he must know when to abstain. In such case he must confine himself to cataplasms, leeches if necessary, and the diminution of drinks in order to check the vomiting, assuaging thirst by pieces of ice, &c. When, however, the inflammation has declined, as indicated by the diminution of the tumor and the flaccidity of the skin over it, he must again have recourse to the taxis. In *enterocele* the taxis has the double object of preventing the establishment of adhesions, and giving the liberated intestine power to resume its functions, which it will do better in the abdomen than elsewhere. For it is a remarkable thing that the intestinal noose remains distended with gas after the inflammation has disappeared, which opposes some slight obstacle to the efforts at reduction. After reduction the gas becomes dissipated over the rest of the canal, relieving this portion of its distension. If a laxative enema were not usually given the stools would yet soon naturally appear. After the reduction we may even leave the patient without a bandage. The hernia projects externally, but without tension, pains, or resistance to reduction. It has returned to its normal state. In *epiplocele* our only object is to prevent the formation of adhesions, which take place much more rapidly than in enterocele; and, as they are especially organized at the fundus of the sac, they not only impede the eventual reduction of the hernia, but, by maintaining the pedicle of the omentum within the ring, render the escape of a portion of intestine much easier, and the application of trusses more difficult. Thus, when the first attempts at reduction have failed, we should treat the local peritonitis by appropriate means, but I always recur to the taxis in a shorter period than in the case of enterocele. If the inflammation is intense and has proceeded on to the suppurative stage, it is remarkable that, in general, false membranes and adhesions cut off the communication between the herniary sac and the peritoneal cavity. The former then resembles an encysted abscess which should be freely laid open, taking care not to destroy these useful adhesions, and then leaving the case to Nature. The same practice is indicated when the abscess is complicated with a fissure of the intestine. If, finally, the inflammation is too extensive to yield to treatment, the case becomes desperate; but at least it is better to let the patient die naturally than to hasten his end by an operation which nothing can justify."—*Gazette des Hôpitaux,* No. 117.

[Perhaps the complication of Hernia by inflammation is too much overlooked in favour of relieving a real or supposed mechanical obstacle. Certain it is that M. Malgaigne in his hospital has successfully treated numerous cases upon the above principles, which, according to ordinary rules, would have been submitted to operation. When we recollect the urgent importance of this being quickly resorted to in the cases for which it is indicated, we see the element of difficulty added by M. Malgaigne's views to a question already sufficiently complicated. It must be borne in mind he limits his recommendation to specified cases, which however are not of unfrequent occurrence."—*Rev.*]

---

### On the Influence which Diseases of the Uterus exert in producing a Faulty Position of the Child.

M. *Tanchou* read a paper in which he endeavoured to show, that although the abundance of the *liquor amnii* or the influence of external circumstances may contribute to the production of the anormal portions of the fœtus in utero, yet that prior or present disease of the walls of the organ is a more probable and the most frequent cause. Would it not, therefore, be proper, after a difficult delivery in which the child has occupied a faulty position, to investigate whether any latent disease of the womb existed, in order that by appropriate treatment the reproduction of this untoward accident might be prevented in future pregnancies. When, however, the anormal position is found to exist habitually in women who have never been ill, and in whom no disordered state of the uterus can be detected, may we not believe the faulty position is due to some primary vicious conformation of the organ, investing its planes of muscular fibres with unequal power and development? M. *Dancel* observed that difficult labours are met with chiefly in large towns in which diseases of the uterus are of frequent occurrence. In provincial towns they are rarely met with, and never among primiparæ in the country. The only difficult labours met with in the country arise in women who have already had four or five children, and have subsequently suffered from diseases of the uterus. M. *Duhamel* considered the question should be reduced to these terms. Is a faulty position of the fœtus more frequently observed in women who have had several children than in primiparæ? If so, a strong probability attaches to M. Tanchou's opinion. He believed in his own experience he had met more examples among women who had had children than in primiparæ: but he also has met with such in the latter. That there should be a larger proportion among the former is in no wise surprising, seeing that there are so many more accouchements of women who have had children than of primiparæ. M. *Guersant* observed that two points required consideration: first miscarriages, and secondly faulty positions. "As to the first, it results from my observation that pathological conditions of the uterus exert a great influence in their production. In young married women they are very frequent, which is attributable to the congested state of the womb. So true is this, that when the female adopts a severe regimen, bodily rest, and other precautions, she avoids miscarriage. As to false positions of the infant, I can say nothing of the effect of the diseases of the womb." M. *Picard* cited several cases in which diseases of the uterus induced miscarriage; but he considered the effect of these in the production of faulty position rather as a theoretical statement than a demonstrated fact.—*Discussion at the Société de Médecine Pratique, Gazette des Hôpitaux,* No. 119.

---

### On Granulations of the Cervix Uteri. By M. Velpeau.

Granulations of the Cervix Uteri are of such frequent occurrence, that we may state it is generally the affection which really exists when ulcerations are said to be present; for, by a medical attendant not accustomed to observe them, they may easily be mistaken. One reason why they are so imperfectly known is that examinations are not made when the woman, as in this affection, does not complain of severe pain. It is a current but erroneous belief that *cancer* is a disease attended with dreadful pain, and if this symptom is so slight as not to excite a fear of it, examination is generally neglected. In fact, cancer may proceed very far with little pain, and most of our patients have explained their not applying for advice at an early period by this circumstance. Some women, however, suffer dreadfully from cancer, and it is from such cases the pictures in books have been drawn; and most suffer greatly towards the termination of the disease, when probably the broad ligaments and nervous plexus become involved.

As to the numerous *varieties of ulceration,* such as the scrofulous, scorbutic, &c., described by various writers upon the diseases of this part, we have never been able to distinguish them.

The *granular condition of the cervix* is always indicated by a more or less abundant whitish discharge; and, although a lactescent, yellowish, discharge, sometimes tinged with blood, may certainly be a symptom of some other diseases, it generally indicates granulations, especially when accompanied with pains in the bowels, groins, and loins, and by loss of flesh. When an examination is made by a *sufficiently-exercised finger,* the cervix is found slightly enlarged and imparts a sensation of granulation to the finger, just as if a raspberry were spread out into a firm membrane. The granules are easily recognized by one accustomed to search for them, and more so by the finger than by the speculum, which, unless in very experienced hands, may lead to error. When this instrument is introduced it raises the mucous membrane of the vagina into two folds, which may easily be mistaken for the cervix, or it may only discover one side of the cervix, and that the healthy one. Of the two means, then, the finger is preferable to the speculum, but each may be advantageously used to controul the results obtained by the other. Sometimes the granulations are disseminated, at others, assembled together in patches. Generally they occupy the os, but at other times they are found within the cavity of the cervix. It is in this last case that the speculum is chiefly useful, as, on exerting a little pressure when the neck is engaged in its extremity, the orifice is somewhat expanded; and, if we then see muco-purulent fluids flow out, granulations certainly exist. Granulations only affect the mucous membrane. The epithelium is destroyed, and the papillary layer denuded, but the proper tissue of the organ remains unaffected. It is seldom that we find any marked tumefaction present. Frequent as is the disease, it presents no other varieties. It is true that ulcerations are sometimes produced by excoriations of the granulations, but they are hardly worth notice, and change nothing as to the nature of the disease. A very important question presents itself—Are granulations dangerous? Some practitioners reply to it affirmatively, believing them as the departing point of cancer: but this has never been demonstrated. Such a number of women have had granulations for a long period without cancer ever resulting, that it is doubtful whether it ever so arises. Granulations, indeed, admit of cure in almost every case, certainly in 19 out of 20—*cauterization* being the means par excellence. The nitrate of mercury is the most efficacious, as well as the most convenient caustic, and never does any mischief. A small pencil moistened with it should be applied to the granulations once a week for five or six weeks, but not for a longer period; inasmuch as, if we prolong the use of the application, the little ulcers it induces are prevented healing. A few baths and some tonic medicine complete the cure.

The history of granulations is much connected with that of *deviations* of the uterus, the two affections so commonly occurring simultaneously. Prior to our investigations, some twenty years since, these deviations were not acknowledged, except as connected with pregnancy; but they are now known to constitute the commonest lesion of the female genital organs, although they are yet frequently mistaken and treated for other affections. Not only may there be anteversion, retroversion, or lateral version of the cervix uteri, but likewise an inflexion of the womb upon himself; and inflexion is of far more common occurrence than inversion. In inflexion we find the cervix in nearly its natural position, while the body of the organ is bent forwards, backwards, or laterally. The cervix is found normal, or nearly so, and as a lump, sometimes tender, can be felt in the pelvis, the case is set down as one of engorgement, and the patient submitted to injurious confinement and medication. Of 50 cases of pretended engorgments at least 45 are examples of deviation only. When the true nature of the case is ascertained, it is evident that we must rely chiefly on mechanical support; and, although this cannot always be effectually applied, we are at all events able to prevent the patient suffering from needless fears, and encourage her to take exercise and good diet, which are two important points. We do not deny that engorgements may exist, but we maintain that they are very rare; and, considering diseases of the uterus in the order of the frequency of their occurrence, we place granulations of the cervix first, then deviations—affections which are met with every day: after which come anormal productions, such as fibrous tumours, cancer, engorgements, &c."—*Gazette des Hôpitaux,* No 117.

---

Clinical Researches upon the Organic Causes and the Mechanism of the Production of the Affections termed Hysterical. By Ch. Schutzenberger, Professeur de Clinique Interne á la Faculté de Strasbourg.

M. Schutzenberger has recently published an interesting series of papers upon the etiology and pathology of Hysteria, illustrated by several cases. We have only space to quote his conclusions, which seem to us to establish more precision in the distinction of the various causes of this distressing malady than heretofore prevailed.

1. The term "Hysteria" has historically two significations: the one symptomatical, the other etiological. 2. Under the symptomatic point of view, it is any thing but a rigorously determined pathological condition; for, if all our authors proclaim the extreme variety of its phenomena, some more especially confine these particularly to more or less general convulsive attacks, while others extend them to nearly all the nervous disturbances observed in women: so that, in practice, the symptomatic and purely nominal diagnosis is often a deception, no one knowing exactly what is to be understood by hysteria. 3. Examined under the etiological aspect, hysteria is in no wise more exactly specified. If there is want of agreement as to the symptoms, there is still more in respect to their cause. Admitted only by induction, and not being generally capable of a practical diagnosis, the etiological expression of the disease possesses scarcely any scientific precision, save in appearance. 4. Under these circumstances we should absolve ourselves from a *specification more nominal than scientific*, which impedes free investigation, and study the nervous functional disturbances less as nosologists who seek out the varieties of a given and known disease, than with the independent spirit of the clinical physiologist.

5. From a first series of facts investigated in this manner it results:—A. That certain local nervous excitements, generally continuous, may become the organic cause of intermitting functional disturbances—exhibiting themselves under the form of more or less general convulsive attacks, with or without the loss of sensibility—without the central organs of the nervous system in general being the subjects of any permanent pathological condition. B. That, among women, *excitement of the ovaries* is the most frequent cause of this kind of disturbance, producing it in a manner analogous to other reflex action. C. We recognize clinically the reality of this cause, since deep-seated pressure will induce local pain and reflex convulsive action. D. Other local irritation may produce analogous phenomena, and an attentive examination may discover such centres of irritation. E. These local irritations, capable of propagating excitement, when simple, and especially that of the ovary, are affections of slight gravity, unless they have been long neglected, or are conjoined with incurable organic conditions. F. In practice, it is of the first consequence to determine the cause of the local nervous excitement. G. As regards the ovary, it may depend on congestion, on inflammation, on degeneration, or on a purely neuralgic condition. H. Our indications of treatment are—1, to remove the determining cause of the local excitement when it is appreciable; and 2, to directly diminish the excited condition of the nerves of the part which form the focus whence the local irritation is propagated to the system. I. The means of effecting the first of these are as various as are the causes themselves. J. Certain substances, and especially assafœtida, castor, and galbanum, seem to exert a sedative effect upon the ovarian excitability; but their employment in no-wise excludes the application of other agents derived from general therapeutics. K. The intermittent nervous excitement or convulsion demands but secondary or palliative measures, ceasing, as it does, when the local irritation is relieved: unless, indeed, under the influence of the frequent repetition of the propagated pathological condition, a morbid degree of excitability of the spinal marrow does not become *secondarily* established, and henceforth capable of being induced by simple physiological stimuli. Although this is only consecutive to simple local irritation, propagated by reflex action, it now becomes an entirely new pathological condition.

6. A second series of clinical researches authorizes the reference of a great number of functional disturbances occurring in the sensitive sphere, to a special *pathological condition*, whose material element is unknown, but which is characterized dynamically by an *exaggerated excitability* of the sensitive nerves. The term *hyperæsthesia* may be used to characterize this condition. A. We may clinically recognize the existence of this organic condition, when *physiological stimuli* or *slight causes of excitement* produce functional manifestions in the sensitive nerves which appear *spontaneous* or *exaggerated.* B. This organic condition of the nervous system is sometimes idiopathic, a part of an original constitution, or it may become developed under the influence of a neglected hygiene. In such cases, too, hygiene offers our most valuable therapeutical resources; for medicines employed with the direct view of diminishing the general excitement merely procure a temporary relief, and the treatment of local excitement can only be regarded as symptomatic and palliative. C. At other times, the morbid excitability of the sensitive nerves is the consequence and effect of *simple* or *chlorotic anœmia.* It is indeed an exaggeration which yet contains much truth, to say that chlorosis rules over the entire nervous pathology of woman—that hysteria is but a species of chlorosis, or as Sydenham expressed it, chlorosis is an hysteric affection. Here, too, we must attack the cause, and *iron* is our sovereign remedy, all attempts at directly reducing the excitement being mere palliatives.

7. A third series of cases reveals the existence of a more complex pathological condition, in which the hyperæsthesia is associated with a particular morbid condition of the spinal marrow, unknown as regards its material element, but dynamically characterized by a *pathological excitability,* in virtue of which the reflex property of the organ becomes exaggerated: so that we may with propriety term this *reflex excitability.* A. This complex condition is recognized at the bedside by—1, characters already attributed to hyperæsthesia; 2, the existence of a greater or less number of *permanent centres of sensibility, the artificial and mechanical excitement* of which induces with facility reflex movements in the form of convulsive attacks. B. As in simple hyperæsthesia, simple or chlorotic anæmia often plays the part of cause of reflex spinal excitability; but this may also be developed suddenly, or it may be consecutive to the frequent recurrence of simple intermittent excitement, originally due to a local cause. C. In cases of this kind the multiple points of departure of the attacks only play a secondary part, and only furnish palliative indications, the importance of which is in an inverse ratio to the multiplicity of the centres of peripheric excitement. D. The fundamental indication consists in fundamentally modifying the organic conditions which incessantly revive the functional disturbance; the hyperæsthesia on the one hand, and the spinal reflex excitability on the other. E. The first of these we have noticed already; but we know of no means of directly acting upon the latter. Blood-letting is usually ineffectual, may prove in many cases injurious, and is really only indicated in those exceptional examples in which the spinal excitability is connected with local congestion or general plethora. Narcotics exert no durable influence; and antispasmodics, such as valerian, assafœtida, castor, &c. are not more useful. The metallic oxides and sulphate of quinine have not been sufficiently experimented upon in these special cases. The means which we have hitherto found most efficacious is the application of cold, either by means of lotions or baths. It is a plan, however, which must be employed cautiously. It is certain also that the exertion of the will may, to a certain point, triumph over this spinal excitability; so that voluntary motions *methodically* practised form one of the best means of preventing the reproduction of the reflex ones. As a principle, we may state that the reflexibility diminishes in proportion as the influence of the will over the spinal marrow is strengthened, and *vice versâ.*

It results from what has now been stated that the well-proved presence of *general excitability* renders the prognosis serious. Although a cure be not impossible, it can only result from a well-digested plan of treatment pursued for a long period.—*Gazette Medicale,* No. 43.

### On the Employment of the Iodide of Potassium in the Treatment of Syphilis.

This recently formed the subject for which the Paris Society of Medicine offered its gold metal. M. *Gibert* was appointed to report upon the merits of the competitors, and he awarded the medal to *M. Payan of Aix*, recommending, at the same time, a silver medal for an essay by M. Bassereau. M. Gibert's Report and M. Payan's Essay are both published in the *Revue Medicale;* and some notice of them will prove a useful *pendant* to Mr. Ormerod's observations upon the same subject, at p. 74 of our present number.

M. *Bassereau's* paper is chiefly valuable as containing an ample detail of M. *Ricord's* experience in the use of Iodine, he having been one of those who have employed it most extensively. According to M. Ricord there is but one *primary* symptom, chancre; and the shorter the duration of this is rendered, either by an early resort to caustic, or a later employment of mercury, the less are secondary symptoms to be feared. Secondary syphilis may be divided into two epochs. During the first, *secondary* syphilis properly so called, those of a superficial character chiefly occur, such as exanthematous eruptions, patches on the mucous membranes, and superficial ulcerations at the mucous orifices. The deeper-seated symptoms, which occur later, such as deep-seated tubercle and serpiginous ulceration of the skin, deep ulcer of the fauces, periostitis, &c. are termed *tertiary*. Between these two classes there is found a mixed or *transition* series, comprehending certain of the pustular and tubercular syphilides, venereal sarcocele, &c. For the primary symptoms, mercurials; for the secondary and transition periods mercury, either alone or combined with iodine; and, in the tertiary period, the iodide alone; are the means recommended by M. Ricord.

M. *Gibert* takes the opportunity of expressing the opinion which a long employment of this remedy has enabled him to form. 1. The Iodide incontestably merits the reputation it has gained as an anti-syphilitic. 2. It may be given in a variety of fluids, but is best administered in distilled water with a little syrup; the quantity varying from 15 to 30 grains per diem, taken in two doses. 3. It succeeds alone in cases of secondary and tertiary syphilis. 4. Its harmlessness renders it especially valuable in syphilitic cachexia, also for women, children, the delicate and feeble. 5. As being more certain and efficacious, and as innocent, Mr. G. prefers the *sirop de deuto-iodure-ioduré*, i. e. iodide of potassium in combination with bi-iodide of mercury. 6. Iodide of potassium is the remedy *par excellence* in those cases in which mercurials fail, while reciprocally these will cure cases which resist iodine.

M. Payan's Essay is the most elaborate exposition of the advantages of Iodine we have yet met with; illustrated, as it is abundantly, not only by cases which have occurred to the author, but by reference to a great number of others which have already been published. He passes the various preparations of iodine which have been recommended under review, and considers that the *iodide of potassium* is in every respect the one to which preference should be given. Its easy *solubility* allows of its being administered at any degree of strength, and in almost any vehicle or combination. It is not *irritating* like iodine and so many of its compounds are, and never gives rise to the accidents which they do, even when given at the extreme ages of life. It never induces *marasmus* or *wasting* of organs or tissues, like iodine frequently does: acting instead as a corroborative or tonic, although at the same time it exerts a remarkable resolvent effect upon pathological productions. If iodine has of late years been successfully employed in a variety of morbid conditions, against which it was formerly pronounced inoperative, this is entirely due to this form of it being substituted for those formerly in use. M. Payan does not approve of the association of iodine with the iodide.

*Mode of Administration.*—Dr. *Wallace* of Dublin (the first practitioner who employed this drug in syphilis to any great extent, and whose clinical lecture in the *Lancet, March* 1836, is frequently quoted from by M. Payan) dissolved ʒij. of the salt in 8 oz. of water, and gave the patient a table-spoonful four times a day. M. *Ricord,* a great authority upon this subject, considers that most cases require about 21 grains per diem as a minimum, divided into three doses. Four or five days after, this dose is gradually increased until double the quantity is taken, which for most cases suffice, although some few may require the maximum, a drachm and a half per diem. M. Ricord, for patients who can afford it, prescribes also syrup. sarsaparilla 500 parts, iod. pot. 16 pts. M. *Lisfranc* commences with 15 grains per diem, and gradually increases the dose until sometimes as much as 120 grs. are taken—the medium quantity being from 45 to 60. M. *Payan* gives the medicine in a *tisane,* or if the patient can afford it, in sarsaparilla. If there are any hypersthenic symptoms, he commences with 8 grs. per diem, and otherwise with 12 or 15, increasing the dose by four grains every few days, until from 30 to 40 grains are reached, beyond which he seldom proceeds. The entire quantity which may be required for the cure of the disease varies much in different persons, some requiring very strong doses, and others being cured just as quickly by very moderate ones. The medicine will be usually required for from one to two months, for the cure of primary symptoms: from two to three for secondary syphilis, and from two to four for tertiary symptoms. If the medicine is given in *pills,* it gives rise to very severe griping, which it never does in solution. When the stomach is very irritable, it may be given in an *enema.*

The author first demonstrates, from his own and others' observations, the immense utility of this drug in *tertiary syphilis.* This class of cases is precisely the one in which mercury has been often found of so little avail, or even to lead to exacerbation of the evil; so that many persons have been accustomed even to attribute the existence of the symptoms to its use. It is evident that a long continuance of the syphilitic diseases impoverishes the system, producing pallor, wasting, &c.; and yet we give one of the most hyposthenic of remedies, possessing the power of attenuating the blood, and diminishing its plasticity. How often have practitioners regretted the absence of a remedy combining the power of a specific and a reparative or tonic! The iodide accomplishes these purposes in the most effectual manner; and very remarkable it is, that while mercury is less efficacious in proportion to the inveteracy of the disease, the rapidity and completeness of the operation of iodine is proportionate to the prior long-continuance of the disease. "For those who might charge us with exaggeration in our appreciation of this invaluable medicine, we may refer to its success in the obstinate cases we have detailed. We have seen it almost instantly arrest the course of the disease; relieve and cure those dreadful pains in the bones which had caused so much misery; disperse exostoses, periostoses, and those gummy tumours which had offered such resistance; cicatrize those terrible, gnawing ulcers; triumph over muscular contractions, and cases of caries and necrosis heretofore attended with such terrible consequences: and, in fact, cause the disappearance of those diatheses which were formerly deemed utterly incurable, the grave holding out the only prospect of escape from them. If, again, we consider that the progress towards cure has always proceeded with unhoped-for rapidity; that the medicine is in itself harmless and exempt from the inconveniences which make so many patients dread mercury, we shall feel less surprize at so favorable an opinion being given. 'I am so persuaded of the efficacy of iodine in tertiary syphilis,' observes M. Ricord, 'that I hesitate not to propose it as a specific in such cases, while it may act as a prophylactic against such, after we have dissipated the secondary symptoms by the aid of mercury.'"

Although most practitioners allow the vast efficacy of this medicine in *tertiary*

syphilis, greater discrepancy of opinion prevails as to its utility in *secondary* syphilis. M. Payan however quotes many cases in its favour, and thus sums up. 1. That, even as regards secondary syphilis, the iod. pot. should be reputed an anti-syphilitic. 2. That it is especially useful in the syphilides. 3. That the longer secondary symptoms have existed, *i. e.* the nearer they approach the category of the tertiary ones, the more obedient are they to the action of this remedy. 4. That this medicine should be especially resorted to when the symptoms have resisted mercury judiciously administered. 5. That we should even commence with it when from the age of the symptoms we judge them removeable by mercury but with great difficulty, or when the debilitated state of the constitution indicates the necessity of reparation. Many cases which have partaken of the character of secondary and tertiary symptoms have benefited by conjoining with the mercurials decoction of sarsaparilla containing the iodide. It would be unjust to deny the antisyphilitic powers of the iodide, because in some cases of secondary or other syphilis it proves inefficacious; for mercury itself, in stages of the disease wherein its beneficial agency is unquestionable, sometimes fails also.

For exemplification of the utility of the iodide in *primary syphilis,* M. Payan is obliged to rely almost exclusively on the evidence his own cases afford, few practitioners having employed it in this stage. He, however, has since 1842 made experiments upon this point, furnishing, he states, highly favorable results. Fifteen of these cases he relates, almost all consisting of indurated chancres, taken, however, unselected, from among the patients who offered themselves to his notice. He concludes—1. That, even in primary syphilis, iodine should not be considered as destitute of anti-syphilitic properties. 2. That it has been found useful sometimes in continuing a treatment commenced with mercury, at others as an exclusive means of treatment. 3. That therefore, without pretending to declare it should in the generality of primary symptoms be preferred to mercurial preparations, the efficacy of which every day's experience testifies, yet there are cases in which it may be highly serviceable. 4. That in cases in which the primary symptoms resist the action of mercury, the substitution of, or the addition of the iodide to the mercury operates as a cure more rapidly than any other succedaneum. 5. There are cases in which we should prefer the use of the iodide to mercury, as when the symptoms have an indolent character, or are connected with a marked hyposthenic condition.—*Revue Medicale,* Tom. II. for 1846 & prec.

---

### On the Employment of Diluents in Urethritis.

M. *Lagneau* read a Report to the Academy of Medicine upon a paper supplied by M. *Loreau,* in which from certain experiments he deduces, the propriety of employing diluents, and abstaining from the use of the *nitrate of potash* in the acute stage of gonorrhœal inflammation of the urethra. The Reporter observes that, although the experiments (made on men) were few in number, they were valuable in consequence of the great care with which they were conducted, and that the conclusions M. Loreau had arrived at were in accordance with the views he himself had long entertained. The copious employment of diluents, it is well known, exerts a remarkable effect in diminishing the *ardor urinæ* of gonorrhœa; but the nitrate of potass which is usually precribed in these cases, according to those experiments, renders the urine eminently acid, and hence more fitting to increase than appease the urethral irritation; while it does not produce a notable increase in the quantity of urine, beyond that resulting from the use of diluents alone. The saline constituents of the urine, which are its irritating portions, become, during the taking the diluents, reduced by one-third of their usual proportion in a given period, while even this quantity is far more abundantly diluted. The drinks seem then to exert a sedative effect upon the kidneys, which then have become rather simple apparatus of filtration than of secretion. Nitre is therefore at least useless, and may become even injurious in this affection, by the acid property it imparts to the urine irritating the inflamed canal. Nevertheless, the Reporter suggests that, when the urethritis puts on an indolent character, or when the secretion of urine is insufficient in chronic cases, the slight stimulus to the canal imparted by the nitre may be useful—viz. in the period of the disease just prior to having recourse to medicinal substances possessing a specific power in arresting the discharge.—*Bulletin de l'Academie,* T. XI. p. 1449.

---

### Fracture of the Cervix Femoris.

In allusion to a case recently occurring in the person of a woman æt. 53, M. *Velpeau* made the following remarks. "Pain and swelling are signs of little consequence, as they may equally exist in fracture or sprain. The impossibility of *raising the heel from the bed* is a sign. It may certainly be present also in a painful affection of the joint; but in the fracture of the cervix there is an absolute impossibility of raising the limb, while in the other affection this may be done if the pain is disregarded. Thus, in a luxation, the patient seems at first unable to raise the limb; but he can do so by perseverance. *Eversion of the foot* is not a pathognomic sign, as it may exist in other lesions; *e. g.* luxation on to the pubes; but in the case of luxation not only is the limb everted, but neither the patient or the surgeon can change its direction, while in fracture the surgeon easily turns the foot inwards. There are other affections in which the foot is rotated outwards, as in paralysis and certain painful affections of the hip. The *admeasurement* of the limb is of great importance, but is of much more difficult accomplishment than is usually believed. The inclination of the axis of the pelvis, or of the limbs themselves, often gives rise to apparent differences, against which we must be on our guard. We must never depend upon mere inspection; but must carefully measure the limb after having placed the patient on his back, and taken care that he lean to neither one side or the other. In these persons, and in those in whom the bony points are prominent, it is easy enough to measure from the iliac spine to the upper edge of the patella; but there are persons in whom the iliac spine is so rounded off that we cannot be certain we are applying the tape upon exactly corresponding points upon the two sides, and an apparent difference, amounting to some lines, may result. So, also, the patella is not only not a fixed point, but its superior angle may be somewhat higher on one side than the other. In this way, several slight errors conjoined may give rise to the belief in a shortening which has no real existence. By carefully guarding against any obliquity of the pelvis, ascertaining exactly the position of the superior anterior spinous process, and carrying the tape down to the malleolus instead of the patella, we shall avoid all serious error."—*Gazette des Hôpitaux,* No. 68.

---

### Treatment of Saturnine Affections.

Two papers have been recently read at the Academy of Medicine (during some of the few intervals of the interminable discussion on the Plague) upon the therapeutical indications in poisoning by lead. The first of these was from the pen of M. *Sandras,* who, together with M. Bouchardat, has been for some time engaged in experiments upon the subject. From these it results that the *persulphate of iron* forms a good antidote to poisoning by lead, copper, corrosive sublimate and arsenic. The researches of modern chemists have shewn that those poisons become deposited in the liver, and MM. Bouchardat, Sandras and

Blondlot have proved the liver to be an organ of elimination. The indication resulting from this is, besides evacuating the digestive canal of the poison, to retain in it an excess of the antidote, with the intention of keeping in an insoluble condition all portions of the lead poison excreted by the liver, until such excretion is completed. To secure the effectual cleansing of the outer and inner surfaces of the body, M. Sandras causes the patient to be placed in a soapy bath immediately after his admission, and then administers croton oil in an enema. This portion of the treatment has been especially prescribed for patients affected with violent colics and constipation. In some cases it has proved of no avail, and has been neglected without inconvenience. About three ounces of the persulphate are dissolved in a pint of water and a tablespoonful of the mixture is given night and morning. Much more considerable doses may be given without any danger, and continued for weeks or months if necessary. To remedy the consecutive accidents resulting from the poisoning, *opium* is given; in small doses for the relief of the colic and cramps, and to procure sleep; and in large doses in tremors with or without convulsions and violent pains in the limbs. Strychnia is given in paralysis with diminution or increase of the sensibility of the skin; and belladonna if neuralgia is present. The regimen should be as substantial as can be borne, During 1844-46, 122 patients, workers in lead, potters, printers, &c., presenting the various marks of lead-poisoning, such as constipation, colic, change of colour of the skin, &c. &c. have been treated in this manner by M. Sandras. Of these, two only have died, one from typhoid fever. All the others were cured, viz. 25 in less than 8 days: 26 in less than 15 days; 17 in less than 3 weeks; 26 in less than 4 weeks; 13 in less than 5 weeks; and 15 in more than 2 and 3 months. There have been two instances of relapse without the patient having again handled lead: and all the others have only been discharged after their cure had been well ascertained. The patients of the first four of these series presented acute symptoms of lead-poisoning; those of the two last likewise suffered from paralysis in different degrees.

M. *Legroux* is the author of the second memoir, and the following are his conclusions. 1. The saturnine affection is a general disease and not a local affection of the digestive canal. 2. The symptomatology of the affection, in spite of the frequent predominance of colic and constipation, which yet may be absent, indicates a disturbed condition of innervation and nutrition. Chemical analysis demonstrates the existence of lead in the organs of patients who succumb, two months and a half after the existence of the disease even, as occurred in a cachectic patient, who died at the end of that period, after undergoing all kinds of treatment. On the other hand, the absorbed lead is eliminated by the skin, as is proved by the formation of sulphuret of lead on the surface of the body after the employment of sulphureous baths, and that several times successively, in spite of soapy baths having in the intervals cleared off the sulphuret already deposited. It results from these facts, that the saturnine affection is a poisoning and not a neuralgia, and that, in the nosological scale, it should be ranged amid the nervous diseases, but side-by-side with the poisonings. 3. The cure of this affection is operated by depuration, and may take place spontaneously. 4. The number and rapidity of the cures obtained in twelve patients treated upon the expectant plan should render us very reserved in the conclusion we draw concerning the value of different modes of treatment. 5. The treatment of these affections comprehends the following indications. A. The destruction of the external cause of the poisoning by the cleansing of the objects and clothes in contact with the body; and the neutralization of the lead deposited on the surface of the skin by means of sulphur baths, which must be insisted upon even a long time after real or apparent cure. B. The destruction of the internal cause of poisoning, by the expulsion or neutralization of the lead deposited on the surface of the mucous membrane, by means of alum, sulphureous waters, sulphuric acid, or the hydrate of the per-sulphate of iron. But these means are prophylactic, not curative. C. The curative indication directs itself to the elimination of the absorbed lead, and acts by inducing or favouring secretion. The cutaneous system may be thus usefully employed, but experience proves that the most prompt results are produced by the use of evacuants, and especially as employed at *La Charité*. D. A fourth indication should consist in counteracting the poisonous agency of lead by a medication possessing an opposite mode of action. According to the Italian doctrine, *opium* would be precisely this agent; but its employment as the basis of treatment in some patients has far from confirmed the good opinion entertained of it. E. The last indication relates to certain morbid conditions which the saturnine affection leaves behind, of which the most constant is a condition of anæmia requiring ferruginous medicines and a supporting regimen. Any plan of medication which does not comprehend the whole of these indications is imperfect. The two first are purely prophylactic, and expectation with them chiefly accomplishes the cure. The third without the others, and especially without the first, exposes to relapse. The fourth, used alone, has the same inconvenience, and the cures so obtained are only the effects of a spontaneous depuration. Finally, without the fifth, the cure would not infrequently leave behind it a more or less serious morbid condition, and a debility which would singularly expose the workman to relapse upon the resumption of his labours.—*Gazette Medicale,* Nos. 38 and 44.

---

### Observations on some Cases of Sudden or very Speedy Deaths, probably dependent on Diseases of the Heart and Large Blood-vessels. By H. C. Lombard, M.D. Geneva.

It is not a very long time since all cases of sudden death were regarded as *apoplexies*, and consequently referred to affection of the brain. At the present time, in consequence of more accurate researches, it is generally agreed, that the greater portion of them depend upon *diseases of the heart or large blood-vessels.* In fact, if we except some cases of hæmorrhage into the Pons Varolii or Medulla Oblongata, it is very rare for sudden death to occur from cerebral apoplexy, the patient almost always surviving the rupture of the cerebral substance, and the compression of the brain by the effused fluid, several hours. We must then receive with distrust several of the cases related under the title of "thundering apoplexy," when the death has been very sudden, and the lesion not verified by an autopsy.

It is quite otherwise with regard to death produced by a morbid condition of the heart and the large vessels. An old practitioner, Dr. Butini of Geneva, once said to me, in reference to a sudden death which took place in a case of heart-disease—"You must not be surprized at this sudden termination. More than a third part of such patients die in this manner, some in turning round in bed, and others, and this is the most frequent case, while getting up to go to stool; so that you must consider sudden death as an ordinary consequence of disease of the heart and hydrothorax." I have had frequent occasion to verify the exactitude of these words, which were addressed to me at the commencement of my medical career: and now, after seventeen years' practice, I wish to add the result of my experience to that of the distinguished physician I have named. In fact, both in my private and hospital practice, I have had frequent occasion to meet with sudden terminations of heart-disease. I have seen patients die suddenly at every stage of these affections; sometimes when the organic changes were so little advanced as not to prevent them following their ordinary occupations; at others, when the extent of the changes, and the complication of dropsy, had long confined them to bed, or at all events to the house: and, if it were desired to establish the proportion between the frequency of sudden deaths among those who seemed only to be slightly attacked and those who were so seriously, it would be in favour of the former I should declare—in other words—*the less advanced the organic disease the more frequent are the sudden deaths.*

Theory easily explains the sudden death of persons suffering from heart-disease. In fact, when the central organ of the circulation is in its normal condition, the temporary cessation of its functions is rarely of serious consequence; while, when its cavities or orifices are in a diseased condition, such suspension, for however short a period, may be attended with the worst consequences. A comparison will cause this to be better understood. Two waggons heavily laden seem to roll along with like facility as long as their movement meets with no obstacle. But suppose the horses stop a while, you perceive a great difference. The axle of one of the waggons presents an irregular surface from lack of oil. That of the other turns easily without noise or friction. The efforts of the horses are now tasked to put the wheels in motion, and while the waggon having the easy axle is drawn with the greatest facility, the other obstinately resists the most vigorous efforts of the horses. The two surfaces, which should slide easily over each other, are motionless, and the horses uselessly exhaust themselves. However trivial this comparison may seem, it may give some idea of what takes place in a diseased heart, which has yet up to a certain time performed its functions without much difficulty. A syncope then however happens to occur, and all the efforts of the cardiac nerves upon the muscular substance, either enfeebled or impeded by obstacles at the orifices, are powerless, and the syncope, which in a healthy heart would have promptly disappeared, becomes the cause of death in a diseased one.

On examining the facts we find these cases may be divided into two quite distinct classes; that in which there is simple syncope and instant death, and that in which there is a considerable impediment in the circulation, and not leading to instant death, but producing such a disorder in the vital functions as leads to their cessation in the course of a few minutes. To fatal syncope are to be referred those cases of sudden death occurring when persons affected with disease of the heart are in the act of turning round in bed, or rising to go to stool. Besides *death by syncope*, however, diseases of the heart and large blood-vessels also frequently induce another description of death which I shall term *death from suffocating spasm*, in order to indicate its predominant characteristic. This, as that from syncope, may occur in persons apparently in the midst of excellent health, but who are really subjects of organic lesions.

Three cases are detailed in exemplification of the characters of this species of death. It is not so sudden as that from syncope, the patient dying in ten or twelve minutes. The attack occurs in the midst of apparent health. The respiration is excessively laborious and noisy, the patient the while tossing his arms in convulsive struggle, and expressing by his countenance or some word, for consciousness continues, the extreme of anguish and terror. A white foam flows from the mouth for some hours after death. "What is the mechanism of death in these cases? Is it a paralysis of the cardiac or inspiratory nerves? Is it a spasm of the cardiac or thoracic muscles? Paralysis, it seems to us, if complete should induce instant death by a fatal syncope; while, if it came on gradually, it would cause a lingering asphyxia which would only induce death at the end of some hours, or at all events in a much longer period than a few minutes; and the pallor of the countenance of these patients contradicts the supposition of the existence of any such asphyxia. I am disposed to place more weight on the explanation by the existence of a *spasmodic condition of the muscles of the heart and of inspiration*, inasmuch as the symptoms are such as those resulting from the condition known as spasm: and the sudden nature of the death indicates the muscles implicated. These cases bear some resemblance to *Angina Pectoris*, but yet need not be confounded with it. Indeed, although death is usually sudden in angina, yet, out of the great number of cases I have perused, and especially those collected by Jurine and Forbes, I have not been able to find one in which it occurred during the first and only attack; while in no one of the three cases I have related had the patient previously suffered from angina. Angina usually occurs either while the patient is walking or asleep; but in two of these cases the patients were quietly seated in their rooms, and no one of them was asleep. Again, in angina there is not that abundant issue of foam from the mouth and nostrils seen in these cases. Lastly, two of the three patients were women; and yet of the 88 cases of angina analyzed by Forbes in the *Cyclopædia of Practical Medicine,* 80 occurred in men and only 8 in women.

"What should be done if called to a case of suffocating spasm soon enough? In the absence of all exact notion of the cause of the affection, we must content ourselves with treating symptoms. I should cause the patient to be seated with his head supported, all ligatures formed by articles of dress being removed. I should apply sinapisms to the thighs, or better still, a cloth dipped in boiling water to the chest. I should administer some alcoholic or anti-spasmodic fluid, and if the patient could not swallow, a napkin might be dipped in æther, Eau de Cologne, or some such fluid, and held near his face. As long as the pulse continued low or the respiration embarassed I should sprinkle the face with cold water. As a general rule I should, on account of the pallor of the countenance, abstain from bleeding."—*Gazette Medicale,* No. 47.

---

### Puerperal Convulsions—Malpraxis.

A woman 25 years of age, of strong constitution and sanguine temperament, was brought while in labour to the Hôtel Dieu, Paris, at *nine* o'clock in the evening. She was in a state of complete insensibility, agitated by convulsive movements, and gave vent to inordinate cries. At *eleven* o'clock the *interne* on duty was called, and found the head presenting, the uterus however having ceased to contract. He determined on turning; but instead of a leg, brought down an arm. After *three hours* ineffectual efforts to rectify this error he left the woman to herself, the convulsions continuing as bad as ever, although bleeding had been twice practised. The child was dead. *Seven o'clock next morning* the woman being in a state of profound coma, the *chef-de-clinique* next tried turning, without success. A surgeon of the hospital was summoned, but declined attending, the case falling within the province of the accoucheur of the hospital. Before the latter could be summoned another *three hours* had elapsed. Turning was then effected; but before delivery was completed the woman had ceased to exist!

A case of puerperal convulsions also recently presented itself in M. Dubois' wards, in which the woman, after suffering an immense number of paroxysms, was delivered by means of the forceps of a living child. Within about thirty hours after delivery she had two other paroxysms; but then seemed in a fair way of doing well, when, after fifteen days of quietude, the convulsions again returned and carried her off, at the end of about the thirtieth paroxysm, the next day.—*Gazette des Hôpitaux,* No. 134.

[With respect to the first of these cases, we have only to observe that the delays and erroneous treatment of the patient reflect the greatest discredit upon the officers of the hospital in which it occurred; and that it is well for them they have no Coroner for Middlesex to visit their neglect with the castigation it deserves. The second case is interesting on account of the remote period at which death took place.—(*Rev.*)

---

### On the Elevation of Parts in the Treatment of some Surgical Diseases. By M. Marc Dupuy.

The object of this paper is to state the great success which has attended the practice long followed by M. Gerdy in the treatment of various inflammatory affections of a surgical character. The influence of position both in the normal and anormal conditions of parts must be familiar to all, and M. Gerdy has but carried out farther than others and more methodically the application of a well-known principle, that the circulation in a part is much aided or impeded according to whether it be performed in accordance with or against the laws of gravity. In inflammation of the *fore-arm* M. Gerdy orders the slings to be worn excessively short, so that the hand of the bad limb may lie upon the opposite shoulder, the elbow being supported in one of the folds of the sling. If the hand is affected the elbow must be laid upon a pad so as to avoid pressure, and the fore-arm sustained in a *vertical* position by means of cushions. The elevation is accomplished in another manner in M. Gerdy's wards with great success, when the patient possesses sufficient docility, viz. by fastening the uninflamed fingers to the rope by which the patient assists himself in turning in bed. The sound fingers must be previously carefully bandaged however, so as to avoid all injurious pressure. If the patient feels fatigued, the rope, which as it were suspends the limb in the air, may be from time to time loosened, taking care however not to allow it to fall into a dependent posture. The means is of an easy application, also, in respect to the *lower extremity*. M. Gerdy raises the end of the bed by placing a chair under it, thus raising the foot upon the summit of an inclined plane. Once so placed, and care taken that no injurious pressure is exerted, the patient must not move from the position even to satisfy natural wants: for he may destroy in a few minutes all the benefits which have been obtained by whole days of repose. Although elevation cannot be so efficaciously applied to the *head* and *trunk* as to the extremities, it yet may be employed to a certain extent. Supposing the *eye* is inflamed, the patient will lie with his head high, and on the opposite side to the one affected. Why are inflammatory affections and discharges from the *womb* so tedious in recovery, but for the stagnation of the blood in the organ? Let a woman, who has been accustomed to keep herself in the vertical posture, go to bed and raise her hips by means of pillows, and she will soon find her case amended. The same principles apply to inflammatory affections of the face, breasts, &c.

The efficacy of elevation is not confined to inflammatory affections, but extends to *hæmorrhages, ulcers*, &c. By attention to it, uterine hæmorrhages may be materially mitigated. So too in M. Gerdy's wards in this way have *varicose ulcers of the legs* been frequently radically cured without any other treatment whatever. In partial dropsy of the cellular tissue the means is efficacious, and thus *œdema of the limbs* disappears which persisted if mere horizontal, or an insufficiently raised position only was resorted to. Even when dependent on disease of the heart, œdema of the feet may be thus dissipated in a few hours. *Hydrocele* and *articular dropsy* have frequently been reproduced by the patient being allowed to go about with the parts in a dependent position. The benefit of position in *varix* and *varicocele* is indubitable, and M. Gerdy feels certain that these troublesome affections might be cured as well as relieved, could it be long enough maintained. *Hæmorrhoids*, which are indeed little else but varices of the veins of the rectum, may be advantageously treated by raising the pelvis while the patient is in bed, the horizontal position not sufficing for disgorging the vessels and relieving the distressing pains. Such persons should not sit on cushions with apertures in them, which do not support the margin of the anus. It follows from what is here said, that in some diseases the elevated posture alone suffices for their cure, and that in many others it is a powerful auxiliary. The paper is illustrated by several cases.—*Archives Generales*, T. xii. p. 295—313.

### Observations on the Use and Abuse of Mercurial Preparations, especially in Inflammatory Affections, By Dr. Sichel.

Administered gradually and for a sufficiently long period, mercury induces the phenomena of scorbutus, that is of an affection of a diametrically opposite character to inflammation, being attended with a *diminution of the plasticity of the blood.* So important a medicine, given in non-purgative doses, is it, that we should as soon think of practising without the aid of the lancet as without it. It is an error to regard it as stimulant on account of the slight temporary irritation it causes. Nevertheless, in treating phlegmasiæ, we should avoid those of its preparations which most partake of this character.

The *antiplastic* agency of mercury is also exerted upon other diseases, which, without being strictly inflammatory ones, depend upon a too great plasticity of the fluids and a too energetic reproduction. This is the case with *syphilis,* upon which, in our opinion, mercury exerts no *specific* effects, but acts only by impoverishing the blood. As, however, this disease is chronic, and often inveterate, and insinuates itself into the different systems in a manner in which inflammation does not, we should employ the most active preparations of the metal which are not so well adapted for the treatment of mere inflammation. Independently, too, of their antiplastic action, they exert a beneficial effect by stimulating the absorbent system. In chronic diseases, active mercurials should be administered for a long period in small doses, while for acute diseases medicines are required of which the dose may be sufficiently increased to ensure the prompt production of the required effects. These more active preparations (such as the deutochloride, the iodides, the red oxide, &c.) are also indicated in other non-syphilitic chronic diseases, dependent upon a slow phlegmasia or an excess of reproduction. Nothing can be more useful than their employment in certain tumours of the soft or hard parts, and some impetiginous or scrofulous affections which seem to verge on cancer. In this last case mercury, combined with iodine, is, of all means, the most efficacious, when the disease has not yet passed the stage in which we sometimes see it arrested and even disappear during the atrophy of the organ affected. In the eye, at least, we have several times seen, under the influence of these two medicines, encephaloid terminate in atrophy of the globe, without relapse or loss of general health.

If in treating inflammation and chronic disease we give mercury in doses which excite the gastro-intestinal membrane we lose some of its most important effects, owing to its not remaining long enough within the economy to exert any direct action upon the blood.

A substance so energetic must necessarily be a dangerous one when administered by an inexpert hand. We may advert to the principal *cautions* which should be observed. 1. The *diet* must be in no-wise stimulant and as little nourishing as possible. If this is not attended to, the plasticity of the blood becomes augmented. 2. All notable change of atmospherical *temperature* should be avoided. Unless this rule is observed, numerous disappointments will occur, and premature salivation is especially likely to be induced. 3. It is a general law that the *special physiological action, or the toxical effect, of a medicinal substance only manifests itself after its action upon the pathological condition* has become exhausted. The operation of this law is well seen in the employment of narcotics in those affections of the venous system which afford distinct indications for their use, as neuralgiæ and tetanus. This last, we know, demands large doses of opium, but the point of saturation must be carefully watched; so that the drug may be laid aside when the precursors of narcotism begin to replace the tetanic symptoms, unless we wish to see, as I have often seen in the hospitals, the patient cured of the tetanus to die of the poisoning by opium. The physiological action of mercury is exerted upon the salivary glands, and with the earliest precursory symptoms of salivation the blood has already lost some of its morbidly plastic character. It is indeed remarkable to what an extent acute inflammation becomes relieved upon the appearance of the precursors of salivation, and how long these are in making their appearance in intense and essentially exsudative inflammations, as iritis, peritonitis, and especially puerperal peritonitis. In this last we are sometimes surprised at finding the abdomen, which the evening before could not endure the weight of the clothes, supporting next day firm pressure with the hand—the precursory symptoms of salivation having manifested themselves in the interval. These are, indeed, the signs of the system having become sufficiently saturated with the mineral, which must be left off as soon as they appear, our object not being, save in very rare and obstinate cases, to excite actual salivation. Instead of then pushing on the mercury, if the disease does not yield, we must, in the case of inflammation, have recourse to other antiphlogistics, and, in the case of syphilis, to iodine, sudorifics, &c.—carefully limiting the regimen, and avoiding exposure to cold. When, however, the precursory symptoms are dissipated, and the disease has yet not yielded, we may recur, again and again, to the mercurial. In syphilis this is almost always necessary.

It is from the non-observance of the above rules that so much mischief has been caused by this remedy, and so much prejudice has been raised against it. The excitement of profuse salivation is especially mischievous. The anti-plastic action of the drug may, after long use, so diminish the coagulability of the blood as to produce a *mercurial scorbutus,* very difficult to cure. It never occurs, however, until long after the induction of salivation, and may be prevented by withholding the drug on the appearance of the precursory symptoms, and recurring to it only after their complete disappearance, if necessary. *Marasmus* may likewise be produced, especially in children and aged persons, if mercury be employed sufficiently long to induce ptyalism or diarrhœa, or the two conjointly. Calomel particularly, must be given with great care to such subjects. It is not sufficient to withhold it when salivation or purging already exist: but at every visit the condition of the salivary organs and digestive tube must be carefully inquired into. From neglect of this precaution infants often suffer severely from the prolonged use of calomel.—*Revue Medicale,* Nov. 1846.

---

### On Latent Pneumonia. By Dr. Saucerotte.

Latent Pneumonia has only of late years sufficiently attracted notice, and indeed it has only been described with any accuracy quite recently by MM. Sestié, Grisolle and Raymond. No observer, that I am aware of, has endeavoured to attach this condition to any prevailing medical constitution, it having been always explained as an individual and exceptional circumstance. Thus, M. Sestié states that pneumonia would not be latent but for the obscurity of the physical signs, and the negligence of those who should seek them. This certainly explains why *pulmonary phlegmasia* is often unknown; but furnishes no account of the specific nature of the morbid form of disease we have to do with. The important point to bear in mind is, that there is a description of pneumonia which does not follow the ordinary course, or exhibit its usual symptoms; and which *from the very first* tends to run into a state of hepatization. It is a form of disease which certain medical constitutions favour the development of, as I observed in our garrison at Luneville, where it prevailed in 1844 and 1846, while in other years it is never met with there.

The entire series of symptoms which characterize pneumonia may be absent, or so slight as to lead to the referring them to different and slighter causes. In general, however, auscultation and percussion do not betray us; but these in affections apparently so slight and so different are frequently not resorted to, M. Saucerotte thus reviews the cases which fell under his care.

*Precursory Symptoms.*—These were usually ill-developed, referrible to the general system rather than to any particular portion of it. They consisted of lassitude, loss of appetite, shivering, &c.; but these were so frequently of so little amount as to enable the soldier to perform his duties only a short while before extreme hepatization was discovered to exist.

*Symptoms.*—The heat of the skin of the thorax so characteristic of pneumonia was never observed, and the pulse continued of the normal number. Little or no difficulty of respiration prevailed, at least as long as the patient continued quiet. A very distinct and generally pretty extensive dulness on percussion was observed, and bronchial respiration and bronchophony existed. In some cases there was a little crepitation around the hepatized portions of lung. The cough was generally very slight, and sometimes did not exist at all.

*Duration.*—This was very variable, according to the constitution of the patient, the treatment adopted, and many other circumstances. The soldiers often obtained leave of absence during their convalescence; but among those who remained the patient would sometimes struggle against the disease for months before he succumbed.

*Diagnosis.*—Pleurisy is the affection with which it has been most generally confounded, a matter of little consequence considering that the same treatment will relieve both diseases. In pleurisy there is less frequently absence of pain in the side; no elasticity of the walls of the thorax on percussion, while the dulness changes its site according to the position of the patient. At a certain stage of the affection there is œgophony, the absence of respiratory sounds if the effusion is considerable, and thoracic vaulting. The disease cannot for any long period be confounded with phthisis. The various acute and chronic affections of the lungs, whether anterior or inter-current, are sometimes masked by, or mask the pneumonia.

*Prognosis.*—It is a general rule that the more a disease is removed from its regular type, and becomes anomalous, the more dangerous it is: and latent pneumonia offers no exception to this. The prognosis was of the gravest character, especially when the affection had made great progress.

*Anatomical Lesions.*—The soldiers being usually removed when seriously ill, M. S. made but few autopsies. In the cases he examined he found the grey induration with a variety of concomitant lesions.

*Etiology.*—Soldiers exposed to various anti-hygienic influences are especially liable to pneumonia; and in the years 1844 and 1846, the latent form was often met with. It is at the end of Winter and after exposure to cold, that the disease especially shews itself. The uniformity of the dress of soldiers, whatever may be the susceptibility of their pulmonary organs, leads to frequent attacks of pneumonia during the atmospheric vicissitudes so common at the commencement of Spring.

*Treatment.*—The absence of febrile re-action forbad large bleeding. I have usually bled but once, and then resorted to cupping or leeches, passing at once to the use of antimony and the application of a very large blister.—*Gazette Medicale,* No. 50.

### On Epidemic Erysipelas as it prevailed in the Beaujon Hospital at Paris. By Dr. Neucourt.

Prior to January and subsequently to March 1843, a case of erysipelas rarely manifested itself among the patients of this hospital, eighty-four in number: but during January and February it raged as an epidemic, attacking every patient having any breach of surface. The description of the attack may be naturally divided into two periods; during the first of which (10th to 25th Jan.) fewer persons were the subjects of it, but in these it proved exceedingly mortal, while during the second (and a longer period) a much larger number of persons became affected with a much milder form of the disease. It is indeed in accordance with the history of most epidemics, that the persons first attacked suffer most severely; so that we may apply to them the celebrated dictum delivered by Sydenham in reference to treatment—*In morbis epidemicis, væ primis.* The patients so attacked were almost all already in a debilitated condition, and they sank under the influence of the disease as if they were poisoned—the local lesion in fact being the least grave part of the disease. At the same time, an ill-condition of all the wounds in the hospital manifested itself, indicating the necessity of abstaining from operations. In this first series of cases the erysipelas was palish or violaceous, atonic rather than inflammatory, and leaving livid traces after death. Gangrene readily formed. The general symptoms were those indicative of extreme prostration—an absence of thirst being notably associated with a dry, brown tongue. Post-mortem examination revealed no cause of death in the condition of the viscera or blood-vessels.

Only a few individuals became the subjects of this serious form of the disease; but during its *second period* every patient in the hospital suffering from wounds was affected. A blister, bleeding from the arm, or any other slight cause, served to induce it. The local symptoms were those ordinarily characterizing the disease. The general symptoms were very different from those observed in the first cases; for, instead of the dry brown tongue, that organ was observed to be broad and white, or sometimes yellowish: the mouth was bitter and pasty; the thirst frequently intense. Nausea and frequent bilious vomitings, disgust for food, sense of uneasiness at the epigastrium, and constant constipation, were other symptoms referrible to the condition of the digestive canal. These symptoms constituting the *embarras gastrique* of Pinel, the *gastric fever* of Frank, and the *bilious fever* of Stoll, are dwelt upon by the author as of leading importance.

These two conditions, *erysipelas* and *embarras gastrique,* invariably coincided; but M. N. does not pretend to decide which acted as cause, which as effect. Nevertheless, in several cases, the *embarras* preceded the erysipelas, while at the same period a great many other patients presented the same symptoms of disorder of the digestive canal, sometimes occurring idiopathically, and at others united to amygdalites, bronchial catarrh, or sometimes to erysipelas of the face. It would indeed seem to constitute what the ancients termed the epidemic genius of the diseases of the season. Certain it is, that as the *embarras* became increased or diminished, so did the erysipelas; and that the most certain mode of relieving the disease of the skin consisted in attacking that of the stomach.

The condition of the *pulse* was markedly different in the two series of cases; for, while in the first, it was small, rapid, or intermittent, in the second, it was large and full, and rarely exceeding 100—generally below it. Its fulness and softness indicated that depletion was seldom required. One symptom—*cephalgia*—offered remarkable intensity and persistence, and which was equally present when the erysipelas affected other parts than the head. It is well known that cephalgia is a frequent symptom of simple *embarras gastrique;* but it is rarely so intense. The *skin* in the first period was arid, dry, and earthy; in the second, hot but not burning, and covered with moisture. The disturbed condition of the faculties occurring rapidly in the former was little observed, except in the erysipelas of the head, during the latter. Delirium, indeed, in these cases, did not posess the gravity which it does in other acute diseases. It was not connected with meningitis; for some patients who manifested it would certainly not have been cured had it been so; while in one who succumbed, after prolonged and intense delirium, no trace of inflammation, or even of congestion, was discovered.

*Treatment.* 1. *Evacuants.*—The changes which the skin undergoes must be looked upon as a mere epiphenomenon, a symptomatic expression of the disease rather than its essence. The numerous topical applications which have been recommended exert no effect upon its ulterior development, and only a few even influence the progress of the actually existing disease. We have adverted to the marked prominence which the gastro-intestinal symptoms presented, and these were also found in a variety of other affections, and often themselves constituted the entire disease, or complicated other diseases. It is to the relief of disorders of this apparatus that treatment should be especially directed. From remote antiquity, and during successive ages, the evacuant method has been regarded with favour, in the relief of this gastric derangement; and it is upon the effectual use of purgatives and emetics that we must rely in the treatment of erysipelas. During their employment the symptoms gradually yield. If the desired improvement is not obtained, the emetic and purgative must be again and again repeated; failure has resulted from such repetition not having been enforced. What should we think of a person declining to repeat venesection in pneumonia, because the disease had not yielded to the first one? The practice of Desault, Tissot, Frank, Boyer, and that of the older surgeons was founded on the recognition of this disordered state of the digestive organs.

2. *Bleeding.*—Although emetics and purgatives are our primary agents in treating this second form of erysipelas, yet they are not the only ones, to the absolute exclusion of others, such as bloodletting for example. In several cases, where the febrile reaction has been predominant over the gastric disturbance, it was employed before having recourse to evacuants, as recommended by Sydenham and Stoll. It is in phlegmonous erysipelas especially, in which the inflammatory character is much more pronounced, that it is indicated.

In the first period, the intensity of the symptoms and the rapid progress of the disease rendered all treatment nugatory.

3. *Topical Applications.*—A great variety of these have engaged attention at different periods—each enjoying its temporary reputation. Such means have a purely local action, extinguishing the erysipelas merely where applied. But the affection is far from consisting in a mere local lesion. The general condition of the patient must chiefly occupy our attention, for the relief of the cutaneous affection is not the cure of the disease. Topical treatment is therefore merely accessory; but yet there are cases in which it is truly valuable. Thus, in respect to recent wounds, especially on the face or head, if erysipelas occurs, and continues for some hours, the reparative power becomes arrested, and union by the first intention impossible. This unfortunate result may be prevented if we possess a topical application capable of preventing the development of erysipelas, or notably abridging its duration after it has become developed. None of the means hitherto proposed possess the power of preventing its development; but some of them, and among these the *sulphate of iron* according to M. Velpeau, seem to exert an influence on its duration. Certain it is that, like cold water, it much assuages the pains, frequently very severe, of erysipelas.—*Archives Generales,* Tom. xii. pp. 414-456.

(Dr. Neucourt illustrates the various points adverted to in his paper by several cases, which are perspicuously detailed; but we regret that he has furnished no statistical account of the total number of persons attacked, proportion of recoveries, &c. We regard the paper as a very useful one in directing attention to the general rather than the topical treatment of erysipelas, and especially to that perverted condition of the alimentary canal which is equally met with in sporadic cases as in the epidemic detailed. We feel surprised, however, to find that he thinks very lightly of the *nitrate of silver* even as a mere accessory means in the cases he has indicated. Conjointly with appropriate internal medication we have seen it render important services—although these have certainly been exaggerated by some writers.—*Rev.*)

---

### On the Nature of Insanity, and its Treatment by Irrigation and Prolonged Baths. By M. Brierre de Boismont.

M. Brierre de Boismont recently presented to the Paris Medical Society a Report upon a Memoir by Dr. Turck upon the *Nature and Treatment of Insanity.* He comments upon some of the Theories of Insanity which have prevailed. Broussais, as is well known, affirmed that insanity always arises from irritation of the brain (and in this statement he has met with many more supporters in this country than he has for his views on gastro-enteritis), but M. B. asks "who has seen the irritation of these capillary vessels? What are the anatomical characters? Most mad-doctors do not admit the existence of pathological lesions, or only regard them as consequences of the disease. I dissected, with M. Bricheteau, the brain of a madman, who had been the subject of intermittent mania for 12 years, and who died after an attack of acute hydrophobic delirium of 12 days' duration, and we were unable to indicate any appreciable lesion. The same thing occurred with respect to an imbecile woman who, after inhabiting my establishment for 18 years, also died after a third attack of acute delirium. It is endeavoured at all hazards to explain the disorders of the mind by anatomical considerations, even when we are quite unaware of what material change takes place in the brain and nerves for the execution of their functions. Every day we observe the most varied and gravest morbid changes without their exciting any influence in producing a state of insanity. The greater or less afflux of blood can so little be considered as the necessary cause of insanity, that frequently apoplexy induces no change in the functions of the intellect, even at the time that effusion has occurred, or paralysis is present. In the physiological condition, the general circulation (as may that of the carotids by some local malady) may be accelerated without the reason suffering. So far from inducing insanity, the greater afflux of blood to the brain induces sleep and somnolence. M. Moreau has of late endeavoured to revive the above theory, by stating *maniacal excitement* to be the primary modification of the intellect, and essential condition of the manifestation of any description of insanity. But this word *excitement* means an excess of organic action, and while this will induce certain forms of insanity, others are produced by quite opposite causes. Cerebral anæmia gives rise to insane conceptions as well as hyperæmia. If febrile excitement induces cerebral disturbances, we observe such disorders only commence in many cases when the decline of the fever has plunged the patient into a state of sinking. With an excitement represented as always 'identical in itself,' the most varying forms of insanity have all the same point of departure. A woman sees her child perish, she becomes stupified and falls into a kind of imbecility, and are we to refer her case to this maniacal excitement? A man, towards the end of a chronic disease which has reduced him to the last stage of marasmus, is seized with a mild form of delirium, in which he believes he is quitting the hospital and following his occupation. Are we to refer this to the same cause? According to this theory, more or less vigorous antiphlogistic treatment should necessarily be put into force. Now, a man is brought to you in a state of furious delirium, and you bleed him until he becomes calm, and the result will frequently be an incurable dementia. Many monomaniacs are much injured by bleeding; and many others recover from their hallucinations by moral treatment alone; and even M. Moreau, the founder of this variety of the irritation of Broussais, states they are frequently relieved by the *datura stramonium.*

"M. Leuret has not sought the cause of the disease in cerebral changes, but states that it may exhibit itself unconnected with any physical symptom, and that it is but an error of the mind, a purely moral disease calling only for moral remedies—our object being to substitute one impression or passion for another. Attributing insanity to a disease of the mind may appear revolting to spiritualists and excite the smiles of the materialist; but we must admit that the question is not so simple as it seems. Elsewhere I have endeavoured to suggest another theory, by establishing that man's ideas spring from two sources. He lives in the midst of an atmosphere of false ideas proceeding from his senses, from education and the social condition in which he exists, and it is there we must seek for the origin of manias, monomanias, &c. In this way we can understand how certain ideas may become diseased, without the divine breath which animates him being implicated. In this point of view, the beautiful results which have attended moral treatment are intelligible enough. We are, however, far from denying the existence of cerebral change, although this as yet has not become appreciable; but it is but a secondary fact in the doctrine of mental diseases, important though it often is in its therapeutical relations."

M. De Boismont next refers to M. Turck's theory which indicates *the skin as the generating organ of insanity* (!), by reason of the disturbance in the product of the electricity of the body its disordered condition induces. We need not waste time in following the exposure of the fallacies of such a doctrine; but may proceed to state the author's mode of treatment, which, indeed, in a modified manner, and without any knowledge of M. Turck or his fancies, M. Brierre de Boismont has been in the habit of employing with success for some years, and an account of which he furnished to the Academy of Medicine in 1842 and 1846. M. Turck's treatment consists in the *prolonged use of tepid baths.* The bath must be prolonged "during a whole or several days," and, if required, repeated during a period of several months. One girl was cured by a "single bath of ten days' duration." By such treatment M. T. declares he has cured four-fifths of the cases which have come before him.

M. De Boismont, while regarding this statement as exaggerated, yet attaches much importance to the curative agency of prolonged baths and irrigations. His former conclusions upon the subject are here reprinted. 1. *All the acute forms* of insanity, and especially of mania, may be so cured in a space of time varying from 1 to 2 weeks. 2. The treatment consists in the employment of prolonged baths and irrigations. 3. The duration of the baths should be in general from 10 to 12 hours, but it may be extended to 15 or 18. 4. The irrigations of the head by a gentle stream of water should be continued during the entire continuance of the bath, unless the patient becomes composed, when they may be suspended. 5. When the patient has taken from 8 to 10 baths without marked amelioration they must be suspended, to be resumed at a future period. 6. The temperature of the baths should be from 82° to 86° F., and that of the irrigations 60°. 7. Of all the forms of insanity, acute mania best yields to this treatment, then simple acute delirium, delirium tremens, puerperal mania, and melancholy monomania with acute symptoms; but, in several of these forms of disease, the cures are neither so rapid or so permanent as in acute mania 8. Chronic mania with acute symptoms, and chronic mania with agitation, may be ameliorated, but not (as stated by M. Turck) cured by this treatment.—*Revue Medicale*, Nov.

